# Role of endocervical curettage during colposcopy for women with abnormal cervical screening results in predicting cervical intraepithelial neoplasia

**DOI:** 10.6026/973206300220418

**Published:** 2026-01-31

**Authors:** Latifa Akhter, Tahsin Tabassum, Mehriban Amatullah, Walida Afrin, Farzana Nasrin, Kashfia Binte Quasem, Rebeka Sultana, Halima Khanam

**Affiliations:** 1 Department of Gynaecological Oncology, Bangladesh Medical University (BMU), Dhaka, Bangladesh; 2 Department of Medicine, Chittagong Medical College Hospital, Chittagong, Bangladesh; 3 Department of Obstetrics and Gynecology, Bangladesh Medical University, Dhaka, Bangladesh

**Keywords:** Cervical intraepithelial neoplasia (CIN), Colposcopy-directed biopsy (CDB), Endocervical curettage (ECC), endocervical curettage, colposcopy, CIN2+, cervical cancer, gynecological oncology

## Abstract

The problem of accurately predicting high-grade cervical intraepithelial neoplasia (CIN2+) in women with positive screening tests remains a challenge in clinical practice. Therefore, it is of interest to evaluate the role of endocervical curettage (ECC) in predicting high-grade cervical intraepithelial neoplasia (CIN2+) in women with positive screening tests. Conducted at Bangabandhu Sheikh Mujib Medical University, the study included 112 women aged 30-60 with abnormal Pap smears or VIA-positive results. While colposcopy-directed biopsy (CDB) detected 23.2% of CIN2+ cases, ECC in combination with CDB identified 35.7%. Data shows that ECC to enhance detection but with lower sensitivity (38.5%) and positive predictive value (41.7%) compared to biopsy alone. ECC proved beneficial in improving CIN2+ detection in high-risk groups.

## Background:

Cervical cancer is the most common gynecological cancer in developing countries. According to World Health Organization (WHO), approximately 604,127 women are diagnosed with cervical cancer every year, and more than 341,831 die from the disease annually [1, 2]. In 2030, the annual number of new cases and death are projected to upsurge to 700,000 and 400,000, respectively. 3 Advanced cervical cancer is one of the main causes of cancer-related mortality in women because of poor access to appropriate management especially, in low- and middle- income countries. 4 According to Global Cancer Observatory (GLOBOCAN) report, cervical cancer is the second most prevalent cancer among Bangladeshi women (12%) with approximately 8,068 (10.6 per 100,000 women) new cases detected every year and causing 5,214 (7.1 per 100,00 women) deaths [3]. Cervical Intraepithelial Neoplasia is a precancerous lesion characterized by a spectrum of disordered growth and development of the squamous epithelium of the cervix induced by infection by high-risk human papillomavirus (HPV). 6 The ectocervix is covered by squamous epithelium, and the endocervix, including the cervical canal, is covered with glandular epithelium. The premalignant transformations of cells occur mostly at the squamocolumnar junction. 7 CIN may exist as low-grade (CIN1) or high-grade (CIN2 or CIN3) [4]. It has always been a dilemma to accurately distinguish CIN1 and CIN2. Moreover, distinguishing CIN2 from CIN3 is also important because CIN2 has a greater regressive potential than CIN3. 8 CIN can progress to carcinoma in situ and invasive carcinoma if it is not treated at an early stage or if the HPV is able to deactivate the host cellular functions [5]. CIN-1 (low-grade) involves the lower 1/3 or less of the epithelium, whereas the more significant CIN-2 involved lower 2/3 of the epithelium and CIN-3 involved more then 2/3 of the cervical epithelium CIN 2 and CIN 3 together called high grade lesion. Dysplasia becomes cancer when it invades the basement membrane [6].

A large majority of cervical cancer is due to the human papillomavirus (HPV), which is a double stranded DNA virus. HPV can be detected in exfoliated cervical cells or vaginal swab samples from approximately 25%-50% of young, sexually active women [7]. Other established risk factors for cervical cancer and its premalignant lesions include - low socio-economic status, smoking, marrying before age 18 years, young age at the first coitus, multiple sexual partners, multiple sexual partners of spouse, and multiple childbirths [8]. The persistence of high-risk HPV infection at one and two years after the initial infection is highly predictive of lifetime risk of pre-invasive and invasive cervical neoplasia [13]. The integration of the HPV genome into the host genome leads to the expression of two viral oncogenes, E6 and E7, which disrupt the function of tumor suppressor proteins p53 and pRb, respectively. This results in the uncontrolled proliferation of infected cells and the development of CIN. The accumulation of additional genetic and epigenetic changes can lead to the progression of CIN to invasive cancer [9]. Cervical intraepithelial Neoplasia can be diagnosed by screening tests like cervical cytology or cytology combined with HPV DNA test, VIA or test. The cases that are suspicious or positive are referred to colposcopy; colposcopy directed biopsy and pathological examination of the lesions are then performed [10]. The treatment decision is based on a screening test and treatment is provided soon or, ideally, immediately after a positive screening test [11]. Colposcopy refers to the use of a specific instrument, a colposcope, for the real-time visualization and assessment of the uterine cervix, specifically the transformation zone, for the detection of CIN or squamous intraepithelial lesions (SIL) and invasive cancer [12]. A colposcopy examination is subjective. Its accuracy to a great extent relies on the physician's experience and there are large variations between physicians' performances. It is also affected by other factors, such as the number, the size and scope of the lesions, cytological result before colposcopy, high-risk human papillomavirus (HR-HPV) testing result, the number of biopsies under colposcopy, the type of transformation zone, and so on. Moreover, colposcopy has several limitations, such as the limited perspective for the lesions in the endocervical canal especially in type 3 TZ, the difficulty to identify infiltration under the presence of cervical epithelium, and the uncertainty of colposcopy image. Hence, these lead to a quantity of false negatives and poor reproducibility of a colposcopy examination [13]. With this context, this study evaluated the role of endocervical curettage in addition to cervical biopsy during colposcopy for women with abnormal cervical screening results in diagnosing cervical intraepithelial neoplasia from Bangladesh perspectives. The findings of the study could aid healthcare professionals in accurately assessing the severity of cervical lesions and determining appropriate treatment strategies, thereby contributing to the effective management and prevention of cervical cancer.

## Materials and Methods:

This study was a cross-sectional analytical study conducted at the Colposcopy Clinic of Gynecological Oncology Department, Bangabandhu Sheikh Mujib Medical University (BSMMU), Shahbagh, Dhaka our 01 year of period from November 2021 to October 2022. The study population was the women aged 30-60 years who were visual inspection of the cervix with acetic acid (VIA) positive or abnormal Pap's report and referred for to the Colposcopy Clinic of BSMMU, Shahbag, Dhaka for further evaluation. Purposive sampling was used according to the availability of the women who fulfilled the selection criteria. To conduct this study, 112 subjects were enrolled attending Colposcopy Clinic, Gynecological Oncology Department, BSMMU, Dhaka who met the eligibility criteria of the study. Women ≥30 years of age with abnormal cervical screening result (VIA/Pap-smear positive) referred for colposcopy at the Colposcopy Clinic of BSMMU. Pregnant women, History of treatment for CIN, History of surgery, chemotherapy or radiation treatment for cervical cancer. Patients with known HIV infection, confirmed diagnosis of invasive cervical cancer (ICC) at the time of referral, HPV DNA test positive were excluded from this study. Semi-structured questionnaire was prepared for this purpose, which will include all the variables of interest. Relevant obstetric and medical history and clinical information was obtained by preformed structured questionnaire. After obtaining Institutional Review Board (IRB) approval, this cross-sectional analytical study was conducted. A total 112 women were enrolled for the study by purposive sampling method. The purpose and procedure of the study was discussed with the patients. Informed written consent was obtained from the patients who voluntarily agreed to participate in the study and perform endocervical curettage in addition to a colposcopy-directed biopsy. Relevant history was obtained, and thorough clinical examination was done in all the subjects. The binocular colposcope SOM® 52 (Karl Kaps, Germany) was used to visually assess the cervix, including cervix visibility, normal colposcopic findings (columnar epithelium ectopy and transformation zone type), acetowhite changes (none, thin or dense) and Lugol staining (stained or non-stained). The impression of colposcopy included normal/benign, low-grade, high-grade and cancer using the Swede score (Table 1 & Table 2). CDB was performed at the squamous-columnar junction in cases of visible lesions (targeted biopsy). If the colposcopy examination showed no lesions (non-targeted biopsy), random 4-quadrant punch biopsies were taken. All colposcopy, CDB and ECC procedures were performed in the same sitting for each study subjects. Histological diagnoses were considered gold standard and graded as CIN2+ (CIN 2, CIN 3, CIS and ICC) and < CIN 2 (CIN 1, chronic cervicitis, and normal) by a single pathologist. The final histopathological results of CDB and ECC were taken as the final diagnosis. Data collection sheets were used from the women's on variables of interest using the semi-structured questionnaire designed for interview, observation, clinical examination, and colposcopy and ECC findings of the women. For every subject a separate data collection sheet was used. Collected data was analyzed using the latest version of SPSS (v27.0). Colposcopy was conducted with the women's lying on her back, legs placed on stirrups, and buttocks positioned at the lower edge of the table. The examination began with an assessment of the vulva and vagina using conventional methods. Subsequently, a suitable sized bivalve Cusco's speculum was gently inserted for further examination. The table height was adjusted to ensure comfort for both the women's and the doctor. Once any suspicious lesion was identified on the vulva, a speculum was placed in the vagina. Acetic acid 5% was applied for eliciting the acetowhite epithelial response. Satisfactory colposcopy was achieved when squamous epithelium, columner epithelium transformation zone and the entire squamocolumnar junction was visible. The cervix was visually divided into four quadrants using lines drawn from 12 to 6 o'clock and from 3 to 9 o'clock positions. Each quadrant was independently graded as normal (no visible lesions), low-grade squamous intraepithelial lesion (LSIL) suggestive of HPV or cervical intraepithelial neoplasia 1 (CIN1), high-grade squamous intraepithelial lesion (HSIL) suggestive of intraepithelial neoplasia 2 or 3 (CIN2 or 3), or invasive cervical cancer. Any abnormal colposcopic impression was biopsied accordingly. In cases where no visible lesions were observed, an iodine solution was applied to the cervix to enhance the detection of abnormal areas. If the colposcopic examination did not reveal any lesions in any quadrant, random biopsies were obtained at the squamocolumnar junction in four quadrants at the 2, 4, 8, or 10 o'clock positions. Multiple biopsies per quadrant were taken as deemed necessary based on the colposcopic impression. Colposcopically HSIL was diagnosed using the swede score system.

Endocervical curettage to assess the endocervix, a sharp Kevorkian curette was placed inside the endocervical canal. Gentle pressure was applied at its tip, and the curette was moved back and forth along the length of the endocervix while being rotated in a circular fashion to sample the entire circumference of the canal. The extension of sampling to lesions external to the cervical OS was avoided during the procedure to minimize contamination with ectocervical tissue. A rapid spinning motion was exerted when removing the curette from the endocervical canal, trapping all tissue and cellular materials in the curette chamber. In women with severe cervical stenosis, a cytobrush (Cervix-Brush) or Wallach broom was used to provide specimens to evaluate the endocervical canal. At every case of data collection, processing and analysis, suggestion from a statistician was sought and the data collected was rechecked to avoid entry of wrong data and ensure analysis using appropriate statistical methods.

## Statistical analysis:

Statistical analyses were carried out by using Windows-based Statistical Package for Social Sciences (SPSS-27). The descriptive statistics of the study was presented in tables, figures, frequency, and percentage, mean ± SD as per the requirement of qualitative and quantitative variables. Chi-square tests were done to observe the association between ECC finding and high-grade squamous intraepithelial lesion of cervix (CIN2+). Confidence intervals for sensitivity, specificity, positive predictive value and negative predictive value of ECC were estimated by the exact binomial method. The Odds ratio (OR) was calculated to measure the likelihood of CIN2+ based on specific variables. Binary logistic regression analysis was then performed to determine the adjusted Odds ratios (AOR) while controlling for potential confounding factors. The p-value <0.05 was considered as statistically significant.

## Results:

Table 3 shows the socio personal information of the women. Here, nearly two-fifths of the women (39.3%) were within 30-39 years age group and another 32.1% were older than 50 years. The table also shows that little over a quarter (25.9%) of the study population were illiterate while 29.5% had completed secondary school level of education or its equivalent. Most (90.2%) of the women were housewives and majorities (63.4%) of the women had monthly family income within >8,585-1,04,391 Tk. Table 4 demonstrates that out of 112 study subjects CIN2+ was detected by CDB only in 26(23.2%), by ECC only in 24(21.4%) and in total by CDB and ECC in 40(35.7%) respondents. Table 5 compares pathological results for ECC and colposcopy-directed biopsies. Among the study subjects, 8.9% of CIN2+ cases were detected by both ECC and CDB. 14.3% of CIN2+ cases were detected by colposcopy directed biopsy but missed by ECC alone. 35.7% of CIN2+ cases were detected by ECC and CDB in total. The 12.5% of CIN2+ cases were missed by CDB alone but were detected when the CDB was applied with ECC. The findings from the binary logistic regression analysis indicated that the factor most strongly associated with the detection of CIN2+ by ECC was colposcopic visualilzation (p<0.05). The adjusted odd ratio for squamocolumnar junction (SCJ) visualization suggests that participants who had inadequate or unsatisfactory visualization during colposcopy were approximately 2.7 times more likely to be detected as having CIN2+ lesions in ECC only (AOR=2.712, 95% CI 1.065-6.9025). Table 6 shows the diagnostic accuracy of the ECC for detection of high-grade Cervical Intraepithelial Lesion. Table 7 designates efficacy of the ECC for the detection of high grade cervical intraepithelial lesions and above (CIN2+) where it had 38.46% sensitivity and 83.72 specificity. Positive and negative predictive for the ECC were 41.67% and 81.82%, respectively.

**Figure 1:**
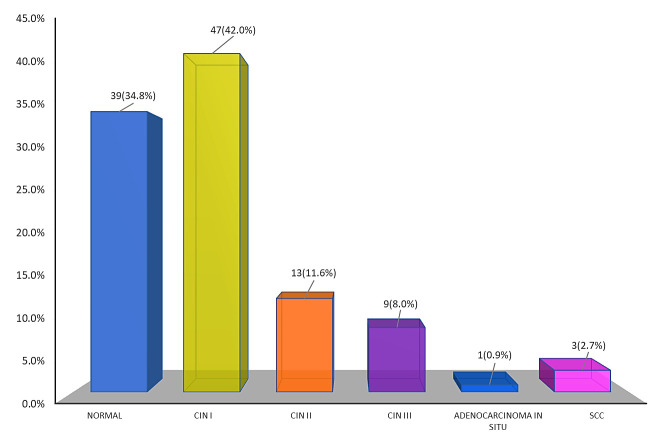
Bar diagram showing the distribution of respondents' histopathological findings obtained from CDB specimens (n=112)

**Figure 2:**
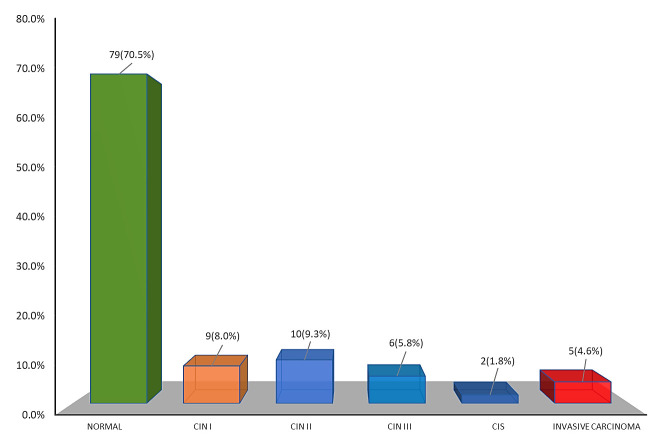
Bar diagram showing the histopathological findings obtained from endocervical curettage specimens (n=112)

Figure 1 shows the distribution of the respondents according to histopathological findings from colposcopy-directed biopsy specimens. Here, CIN I was observed in 42.0%, and normal in 34.8% of the women. CIN II and CIN III were observed in 11.6% and 8.0%, respectively. Only 0.9% of women were diagnosed with adenocarcinoma in situ (AIS), whereas 2.7% had invasive cervical cancer. Figure 2 exhibits the distribution of the study population according to histopathological findings from ECC specimens. Here, the majority of the respondents (70.5%) revealed normal. CIN I was observed in 8.0%, while CIN II and CIN III were observed in 9.3% and 5.8%, respectively. Carcinoma in situ (CIS) was noted in only 1.8%, whereas 4.6% of women had invasive cervical cancer.

## Discussion:

In the present study, the respondents exhibited distinct demographic characteristics, with 39.3% of the patients falling within the 30-39 age group, while 32.1% were older than 50. Educational attainment varied, with 25.9% classified as illiterate and 29.5% having completed secondary school or its equivalent. A significant majority (90.2%) of the women were housewives, indicating a predominant occupation in the sample. Furthermore, a substantial proportion (63.4%) of the participants belonged to the middle-class socio-economic status showed comparable findings, reporting 96.3% housewife, 0.2% labor, 2.3% service, 0.6% businessman, and 0.6% teaching [14, 15]. They also reported two thirds of the families belonged to low- and middle-class income groups. The study conducted by in China observed that individuals in the age group of 46-55 years had a higher risk of developing CIN2+ compared to others [16]. On the other hand, higher education was found to have a protective effect against CIN2+, suggesting that individuals with a higher level of education had a lower risk of developing this condition. In this study, cervical intraepithelial neoplasia grade I (CIN I) was observed in 42.0% of patients, normal in 34.8%, moderate dysplasia (CIN II) in 11.6%, severe dysplasia (CIN III) in 8.0%, adenocarcinoma in situ (AIS) was diagnosed in only 0.9% of women, while invasive cervical cancer was found in 2.7% of cases. Overall, < CIN 2 constituted 76.8% and CIN2+ were 23.2%. In a similar study by Wei *et al.* (2023) observed 80.3% study population on biopsy were < CIN 2, while 19.7% were CIN2+ [17].

Among women in the CIN2+ group, a higher proportion married between the age group 15-17 years (34.1%), while 25.0% reported getting married at or above the age of 21. A higher number of women with CIN2+ had a history of multiple sexual partners (25.0%) compared to those with a single sexual partner (23.1%). The prevalence of post-menopausal women was higher in the CIN2+ group (26.2%) compared to pre-/peri-menopausal women. Women with > 2 children comprised 30.6% of the CIN2+ group, while 20.0% of nulliparous women belonged to the CIN2+ group. Additionally, 24.0% of respondents in the CIN2+ group had a history of using oral contraceptive pills, whereas the majority in the < CIN 2 group did not take any OCP. However, no statistically significant differences were found in the distribution of reproductive and obstetric characteristics between the two groups (p>0.05). Song *et al.* in their study revealed that the average age at menarche was 16.2±1.8 years, and the average age for sex debut was 21.1±2.1 years. The vast majority (92.0%) of the women had contraception measures and 79.7% of them had sterilization. In a retrospective study found that the Chinese women who underwent cervical biopsy for detection of CIN had an average of 3 pregnancies, 2.3 live births, and a average of 1.5 lifetime sexual partners [18]. The histopathological analysis of the ECC samples revealed that the majority of the respondents (70.5%) had normal. Additionally, 8.0% of the samples showed CIN I, 9.3% showed CIN II, and 5.8% showed CIN III. A small percentage of women (1.8%) had carcinoma in situ (CIS), while 4.8% were diagnosed with invasive cervical cancer (ICC). These results highlight the importance of utilizing endocervical curettage as a diagnostic tool for identifying various cervical pathologies, including pre-cancerous lesions and invasive cancer. In the study conducted by, Zhao *et al.* (2025) [19] the findings revealed that ECC was able to detect CIN II or worse in a significant proportion of cases. Out of 364 cases, 98 (26.9%) showed CIN II or worse on ECC. Specifically, of 364 women with CIN II+, 21 were found to have invasive cancer, with ECC detecting 16 (76.2%) women with invasive cancer; 60 of 173 (35.8%) women with CIN III and 20 of 170 (11.8%) women with CIN II.

In addition, logistic regression analysis identified unsatisfactory colposcopic visualization as the most significant independent predictor of CIN2+ detection by ECC. Women with inadequate or obscured squamocolumnar junction visualization were approximately 2.7 times more likely to have CIN2+ detected through ECC compared to those with satisfactory visualization (AOR=2.712, 95% CI: 1.065-6.905; p=0.036). This finding underscores the diagnostic value of ECC, particularly in cases where colposcopy is limited by poor visualization, thereby reinforcing its role as a complementary tool in the detection of high-grade cervical lesions. The reported sensitivity, specificity, PPV, NPV and accuracy of ECC in the present study was 38.46%, 83.72%, 41.67%, 81.82% and 73.21%. This suggests that while ECC alone may have limitations in terms of sensitivity and PPV, it can still provide valuable information in ruling out the presence of CIN2+ lesions. Moreover, it was observed that among the study patients with CIN2+ lesions, 8.9% of cases were detected through both endocervical curettage (ECC) and biopsy. This indicates that ECC played a valuable role in identifying these cases, complementing the findings of the biopsy. Remarkably, 14.3% of CIN2+ cases were detected by biopsy alone but were missed by ECC. However, when ECC was combined with biopsy, a total of 35.7% of CIN2+ cases were detected. This suggests that ECC can enhance the diagnostic yield when used alongside biopsy. Furthermore, it was found that 12.5% of CIN2+ cases were missed by biopsy alone but were detected only by ECC. These findings highlight the importance of incorporating ECC into the diagnostic process, as it can help identify additional cases that may be missed by biopsy alone. Similar findings were reported by Pretorius *et al.* (2004) [20] also exhibited that the endocervical curettage has a sensitivity of 0.38, a specificity of 0.85, a PPV of 0.56, and an NPV of 0.73, which was also consistent to the present study findings.

## Conclusion:

Endocervical curettage is an effective tool for the diagnosis of cervical intraepithelial neoplasia in screening-positive patients during colposcopy. The accuracy and reliability of CIN detection can be improved, facilitating appropriate management and treatment decisions of patients with CI by incorporating ECC.

## Figures and Tables

**Table 1:** Swede score

**Sorec**	**0**	**1**	**2**
Aceto uptake	Zero or transparent	Shady, Milky (not transparent; not opaque)	Distinct, opaque white
Margins/Surface	Diffuse	Sharp but irregular, jagged, geographical satellites	Sharp and even, difference in surface level, including 'cuffing'
Vessels	Fine, regular	Absent	Coarse or atypical
Lesion size	<5mm	5-15mm or 2 quadrants	>15mm or 3-4 quadrants/ endo-cervically undefined
Iodine staining	Brown	Faintly or patchy yellow	Distinct yellow
Total score (maximum 10)			

**Table 2:** Interpretation of swede score

**Overall Swede Score**	**Colposcopy prediction of probable histology**
0 - 4	Low grade / normal CIN I
5 - 6	High grade / noninvasive cancer CIN 2 +
7 - 10	High grade / suspected invasive cancer CIN 2 +

**Table 3:** Distribution of the respondents by their socio-personal information (n=112)

**Characteristics**	**Frequency (n)**	**Percentage (%)**
**Age (in years)**		
30-39	44	39.3
40-49	32	28.6
≥ 50	36	32.1
Mean (±SD)	44.1± 9.67	
**Educational status**		
Illiterate	29	25.9
Up to Primary	25	22.3
Up to SSC/equivalent	33	29.5
Up to HSC/equivalent	14	12.5
Graduate and above	11	9.8
**Occupation**		
Housewife	101	90.2
Service holder and others	11	9.8
Monthly income status (in Taka)#		
Low (≤ 8,585 Tk)	22	19.6
Middle (>8,585-1,04,391 Tk)	71	63.4
High (≥ 1,04,391 Tk)	19	17
#Low income ≤ 8,585 BDT/Month,
middle income = >8,585-1, 04,
391 BDT/Month, and High income
≥ 1,04,391 BDT/Month (World
Bank and UNDP, 2022)

**Table 4:** Distribution of the respondents according to CIN detection by colposcopy directed biopsy and/or ECC (n=112)

**Investigations**	**CIN2+**		**<CIN 2**	
	**n**	**%**	**n**	**%**
Colposcopy-directed biopsy	26	23.2	86	76.8
Endocervical curettage	24	21.4	88	78.6
CDB + ECC	40	35.7	72	64.3

**Table 5:** Comparison of the study population according to their histopathological findings between ECC and CDB (n=112)

**ECC**	**Colposcopy-directed biopsy**		**P-value**
	**CIN2+ (n = 26)**	**<CIN 2 (n = 86)**	
CIN2+ (n = 24)	10 (41.7)	14 (58.3)	0.016^a^
<CIN 2 (n = 88)	16 (18.2)	72 (81.8)	
^a^Chi - square test
Figures in parentheses ( )
represent percentage out of row total.

**Table 6:** Diagnostic accuracy of the ECC for detection of high-grade Cervical Intraepithelial Lesion (n=112)

**Diagnostic Accuracy**	**ECC**	**95% CI (Lower - upper)**	**P-value**
Sensitivity (%)	38.46%	20.23% to 59.43%	
Specificity (%)	83.72%	74.20% to 90.80%	
PPV (%)	41.67%	22.11 to 63.36%	0.016^a^
NPV (%)	81.82%	72.16% to 89.24%	
Accuracy	73.21%	64.02% to 81.14%	

**Table 7:** Efficacy of the ECC for the detection of high grade cervical intraepithelial lesions and above (CIN2+)

**Attribute**	**Coefficient (B)**	**S.E. (B)**	**AOR+**	**95% CI for OR**	**P-value**
Age (<50 years)	0.6818	0.5164	1.9759	0.718-5.433	0.1879
Age (≥50 years)	0	0	0	0	0
Colposcopy Visualization	0.9988	0.4772	2.7122	1.065-6.905	0.0366
Satisfactory	0	0	0	0	0
Unsatisfactory	0.9988	0.4772	2.7122	1.065-6.905	0.0366

## References

[1] https://iris.who.int/.

[2] Singh D (2023). The Lancet Global Health,.

[3] Kussia B (2024). BMC Cancer..

[4] Tekalign T (2022). PLoS One..

[5] Uddin AFMK (2023). South Asian J Cancer..

[6] Liverani CA (2016). Eur J Cancer Prev..

[7] Balasubramaniam SD (2019). Medicina (Kaunas)..

[8] Chen R (2023). Diagn Pathol..

[9] Small W Jr (2017). Cancer..

[10] Sellors JW, Sankaranarayanan R (2003). World Health Organization..

[11] Brown DR (2013). J Infect Dis..

[12] Kashyap N (2019). Asia Pac J Oncol Nurs..

[13] Goodman A. (2015). BMJ..

[14] Pal A, Kundu R (2020). Front. Microbiol..

[15] Khanam A (2028). Bangladesh Journal of Obstetrics & Gynaecology..

[16] Tao L (2014). BioMed Central Public Health..

[17] Wei B (2023). BMC Womens Health..

[18] mSong Y (2017). Oncotarget..

[19] Zhao YQ (2015). BMC Cancer..

[20] Pretorius RG (2004). Am J Obstet Gynecol..

[21] Van J (2015). J Low Genit Tract Dis..

